# Supplementary material for: Stacking fault energy in concentrated alloys
Source: Nat Commun. 2021 Jun 11;12:3590. doi: 10.1038/s41467-021-23860-z (PMC8196205; doi:10.1038/s41467-021-23860-z)
Supplement: Supplementary file 1 — Supplementary Information [file 41467_2021_23860_MOESM1_ESM.pdf]

SUPPLEMENTARY FIGURES:

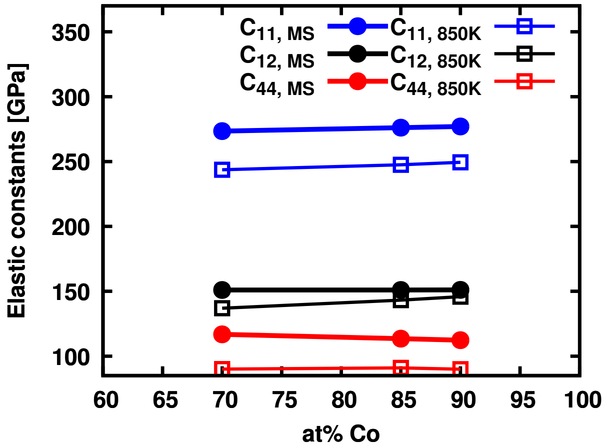

Supplementary Figure 1. Finite temperature elastic constants and the zero-temperature Molecular Statics elastic constants of the model NiCo alloys. The finite temperature elastic constants are obtained using the fluctuation theory under constant stress condition. All finite temperature elastic constants were lower than the zero-temperature counterparts. Each of the simulations were carried out for a total of 10 ns. The first 1 ns is to reach equilibrium, and the final values are obtained by averaging over the latter 9 ns of MD/MC steps.

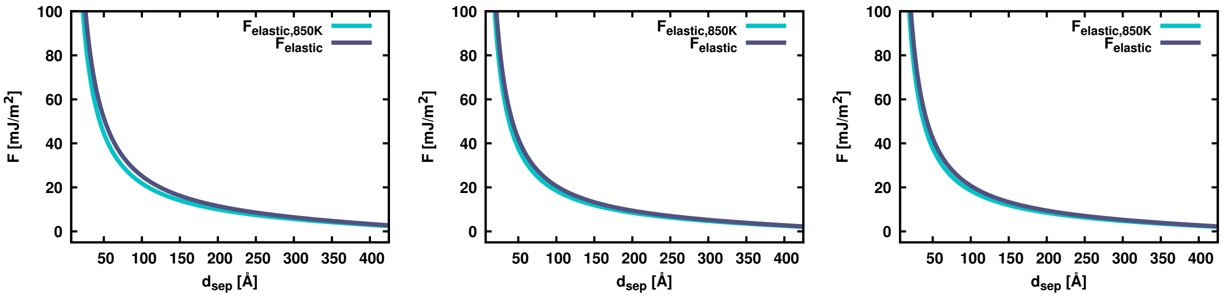

Supplementary Figure 2. The temperature effects on elastic interaction force ($F_{elastic}$) between the partial dislocations. The cyan curves show the $F_{elastic}$ computed with the elastic constants at 850 K. At elevated temperature, there is a steeper drop of the line as we increase the separation distance between partial dislocations, but the temperature dependence of this repulsive force is small to negligible. From left to right are the $F_{elastic}$ curve for positive/zero/negative SFE NiCo alloys, respectively.

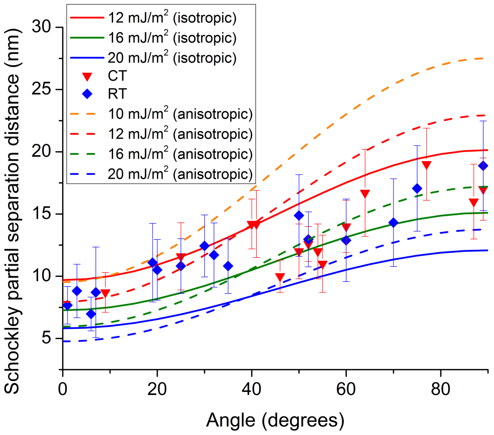

Supplementary Figure 3. The predicted dislocation dissociation distances in equiatomic CrCoNi medium entropy alloy from isotropic and anisotropic elasticity in comparison with experimental measurements. The isotropic and anisotropic elasticity predictions are drawn as solid and dashed lines, respectively. The experimentally measured dissociation distances are at room temperature (RT) condition and cryogenic temperature (CT) condition.

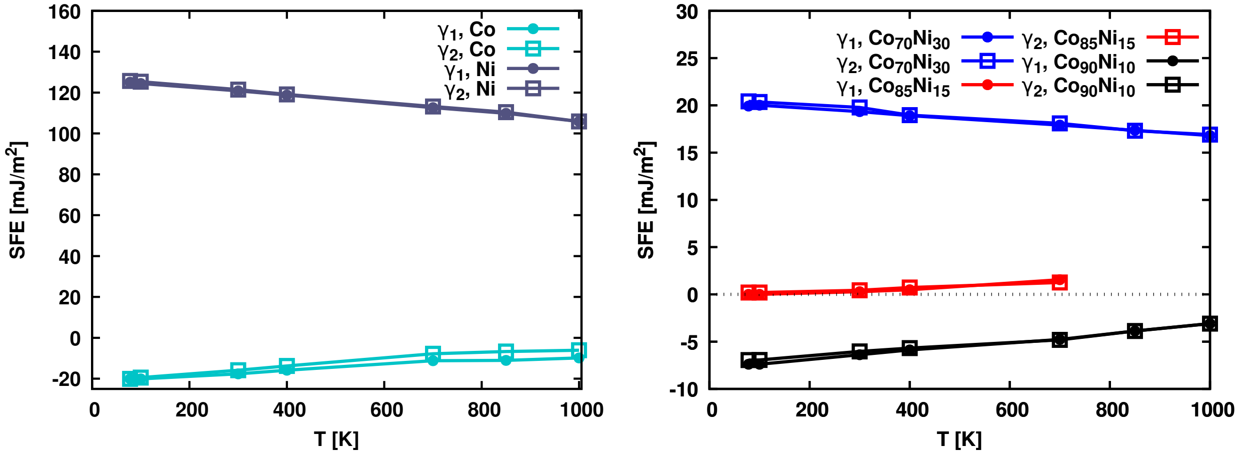

Supplementary Figure 4. The SFE estimation from the first and second order axial-next-nearest neighbor- Ising (ANNNI) model. The ANNNI model utilizes the temperature-dependent free energy of fcc, hcp and dhcp phases, and the temperature-dependent fcc phase lattice constant of the corresponding pure element and NiCo alloy. The SFE values from the first and second order axial-next-nearest neighbor-Ising (ANNNI) models are labelled as γ_1 and γ_2, respectively.

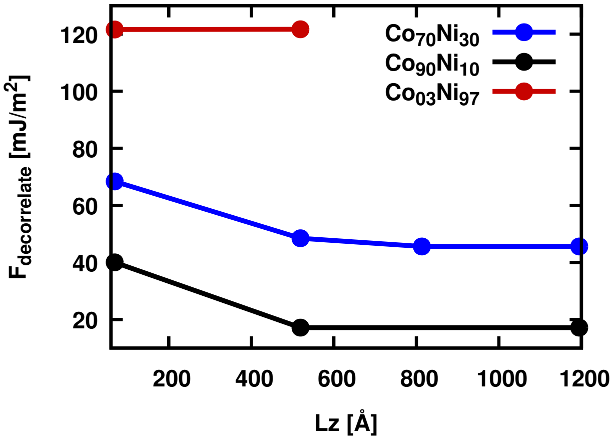

Supplementary Figure 5. Convergence of decorrelation stress vs dislocation line length. In the dilute alloy, the decorrelation force is already converged at 69 Å. A longer dislocation line is required for alloys with higher concentrations. For $Co_{70}Ni_{30}$ and $Co_{90}Ni_{10}$ alloys, the decorrelation force converges at 813 and 518 Å, respectively.

SUPPLEMENTARY NOTE 1: THE ANISOTROPIC ELASTIC INTERACTION OF DISLOCATIONS

The elastic interaction energy between two parallel straight dislocations can be written as:

$$E_{ij} = \frac{K}{2\pi}\mathbf{b}_i\mathbf{b}_j \ln\left(\frac{d_{ij}}{d_0}\right) + \frac{K(\mathbf{b}_i \cdot \mathbf{d}_{ij})(\mathbf{b}_j \cdot \mathbf{d}_{ij})}{|d_{ij}|^2} \tag{1}$$

where $\mathbf{b}_i, \mathbf{b}_j$ are the Burgers vectors of the two dislocations, $\mathbf{d}_{ij}$ is the separation distance between the dislocations, d_0 is the core radius and K is the elastic energy factor [1]. Since we are examining a dissociated full dislocation, $\mathbf{b}_i, \mathbf{b}_j$ are the Burgers vector of the two partial dislocations, and d_{ij} the separation distance between the two partial dislocations. From here, the force per dislocation line between two partial dislocations is simply the derivative of the energy

$$F_{ij} = -\frac{\partial E_{ij}}{\partial d_{ij}} = \frac{K}{2\pi}\frac{\mathbf{b}_i\mathbf{b}_j}{d_{ij}} \tag{2}$$

The energy factor can be obtained using different methods, here we use the sextic formalism,

$$K = \mathbf{b}_i B_{ij} \mathbf{b}_j, B_{ij} = -\frac{1}{2\pi}\int_0^{2\pi} ([mn]_{ik}[nn]^{-1}_{kl}[nm]_{lj} - [mm]_{ij})d\theta \tag{3}$$

where $[mn]_{ij} = m_k C'_{ijkl} n_l$, C'_{ijkl} is the rotated stiffness tensor. Using the elastic constants of the model NiCo alloy and the experimental elastic constants for the CrCoNi alloy, we obtain

the elastic force per unit line of the partial dislocations, discussed in the main text.

To evaluate the effect of anisotropy on experimentally measured separation distance vs the dislocation characteristic angle, we use the force balance equation with the following assumptions. We use the experimental elastic constants ($C_{11} = 249.4, C_{12} = 159.0, C_{44} = 138.4$ GPa) of the CrCoNi alloy from Laplanche *et al.* [2] and assume the dislocation lies on the $\{111\}$ glide plane, with its line direction along $\langle 110 \rangle$. Note that in reality the Burgers vector is fixed and the dislocation line can change its angle with respect to the Burgers vector direction. Since, B_{ij} is calculated for a specific line direction, the assumption of fixing the line direction results in a constant K for all angles and therefore, simplifies the calculations significantly. The same assumption has been made in the literature [3]. Balancing the elastic force per dislocation line with the stacking fault energy gives the equilibrium dissociation distance

$$d = \frac{K}{2\pi} \frac{\mathbf{b}_i(\theta)\mathbf{b}_j(\theta)}{\gamma} \tag{4}$$

where $K = \mathbf{b}_i(\theta) B_{ij} \mathbf{b}_j(\theta)$, $\mathbf{b}_i(\theta)$ and $\mathbf{b}_j(\theta)$ are the rotated Burgers vectors of $a/6\langle 112 \rangle$ Shockley partial dislocations [1]. This is the same as the anisotropic part in equation (1). Note that in reality for an edge dislocation with Burgers vector in the $\langle 110 \rangle$ directions on the $\{111\}$ glide planes, the line direction should be along $\langle 112 \rangle$ directions. The separation distance between the partial dislocations under our assumption and the actual solutions are 27.5, 23.0, 17.2 and 13.8 nm and 26.8, 22.3, 16.7 and 13.4 nm for SFE= 10, 12, 16 and 20 mJ/m^2, respectively. A comparison of the pure edge dislocation using our assumption and the actual solution shows that they are not exactly the same but our approximation is reasonable.

In addition, we compare the dissociation distance obtained from Equation 4 to that obtained assuming isotropic elasticity. Using isotropic elasticity, the dissociation distance d is related to the dislocation characteristic angle (θ) and stacking fault energy γ via

$$d = \frac{G b_p^2}{8\pi\gamma} \frac{2-\nu}{1-\nu} \left(1 - \frac{2\nu\cos(2\theta)}{2-\nu}\right) \tag{5}$$

where G is the shear modulus, b_p is the magnitude of the Burgers vector of the $a/6\langle 112 \rangle$ Shockley partial dislocations and ν is the Poisson's ratio in the isotropic approximation [3]. The shear modulus CrCoNi was measured to be 87 GPa in [4] and is used here. The isotropic elasticity predicts the sfe for CrCoNi alloy to be within 12-20 mJ/m^2. The isotropic and anisotropic elasticity predictions mostly overlap, results in similar predictions except that

the anisotropy predicts a smaller dissociation distance near the screw character dominant side. This is why a smaller sfe is predicted in the anisotropic elasticity.

Next, we calculate the elastic interaction between the Shockley partials in our simulation, considering all their periodic images. Since the dislocation in the simulation is subject to periodic boundary conditions, the simulation is equivalent to having an array of dislocation spaced with L_x intervals in the model NiCo alloy. The elastic force on one partial dislocation from the other dislocations should be

$$\begin{aligned} F_{elastic} &= -\frac{\partial E_{ij}}{\partial d_{ij}} = f(d_{ij}) + f(L_x + d_{ij}) - f(L_x - d_{ij}) + f(2L_x + d_{ij}) - f(2L_x - d_{ij}) + ... \\ &= \frac{K}{2\pi}(\frac{\mathbf{b}_i\mathbf{b}_j}{d_{ij}} + \frac{\mathbf{b}_i\mathbf{b}_j}{L_x + d_{ij}} - \frac{\mathbf{b}_i\mathbf{b}_j}{L_x - d_{ij}} + \frac{\mathbf{b}_i\mathbf{b}_j}{2L_x + d_{ij}} - \frac{\mathbf{b}_i\mathbf{b}_j}{2L_x - d_{ij}} + ...) \\ &= \frac{K\mathbf{b}_i\mathbf{b}_j}{2\pi}\left(\frac{1}{d_{ij}} + \sum_{n=1}^{\infty}(\frac{-2d_{ij}}{n^2L_x^2 - d_{ij}^2})\right) \end{aligned} \tag{6}$$

Convergence of the infinite series $\left(\sum_{n=1}^{\infty}(\frac{-2x}{n^2L_x^2-x^2})\right)$ can be tested using the integral test for p-series, assuming d_{ij} as a constant: $\int_1^{\infty}\frac{-2x}{n^2L_x^2-x^2}dn = \frac{-2x}{L_x}\left(\frac{1}{2x}\ln\left|\frac{nL_x-x}{nL_x+x}\right|\right)\Big|_1^{\infty} = \frac{1}{L_x}\ln\left|\frac{L_x-x}{L_x+x}\right|$. Since the integral converges, so does the sum. The final analytical solution of Equation 6 is:

$$F_{elastic} = \frac{K\mathbf{b}_i\mathbf{b}_j}{2\pi}\left(\frac{1}{d_{ij}} + \frac{-L_x + \pi d_{ij}\cot(\pi d_{ij}/L_x)}{L_x d_{ij}}\right) \tag{7}$$

this is the same as equation (2) in the main test. The energy factor is calculated where the dislocation lies on {111} glide planes, and the line direction is along ⟨112⟩ directions. K is evaluated using elastic constants, computed for each alloy as reported in Supplementary Figure 1.

SUPPLEMENTARY NOTE 2: FINITE TEMPERATURE EFFECTS ON PARTIAL DISLOCATIONS

Since we study the finite temperature effects on the separation distance between the partial dislocations, here we also discuss the effect of temperature on the elastic constants of the alloys. To obtain the elastic constants at finite temperature using MD, we use the fluctuation formalism under constant stress as described by Shinoda *et al.* [5]. Each defect-free fcc cell contains 5120 atoms with periodic boundary conditions in all directions. We carried out each MD simulation for a total of 10 ns. The first 1 ns is to reach equilibrium,

while we averaged the latter 9 ns for the elastic constants calculations. For the alloys, we also implemented Monte Carlo atom swaps at every 500 MD steps. Each time with 100 attempts to randomly pick and swap the atom type of the selected atoms. The swap probability is based on the Metropolis criterion at 850 K. The elastic constants are obtained by averaging over the latter 9 ns of MD/MC steps.

Under NPT ensembles, the elastic constants are related to the strain fluctuations. Here, the fluctuation formula is [6]

$$\delta\big(\epsilon_{ij}\epsilon_{kl}\big) = k_B T (C)^{-1}_{ijkl} / V_0 \tag{8}$$

where $\epsilon = \frac{h_0^{-T} h^T h h_0 - I}{2}$ is the strain matrix, $(C)^{-1}_{ijkl}$ is the compliance tensor, h, h_0 are the instantaneous and average cell vectors, V_0 is the average volume of the cell, and k_B is the Boltzmann constant. We obtain the stiffness tensor using the inverse of the compliance tensor, and hence the elastic constants. The final average of the CoNi alloys' elastic constants are shown in Supplementary Figure 1.

Different elastic constants will change the $F_{elastic}$ equation, which changes the magnitude of the repulsive force on partial dislocations. We also implemented the thermal expansion of lattice constants for the calculations, where the averaged thermal expansion ratio used is 1.0095 at 850 K. In Supplementary Figure 1, the elastic constants decrease with temperature for all alloys. This will result in a steeper line in the force vs separation distance plot. We have plotted the new line to demonstrate the effect in Supplementary Figure 2. Finite temperature shifts the $F_{elastic}$ curve such that the equilibrium separation distance should decrease slightly. But the magnitude is very small and will not alter the main argument in our paper. We thus only show the 0 K $F_{elastic}$ in Fig. 4.

Other than elastic constants, SFE is the other relevant parameter that can be temperature-dependent. We have discussed the effects for the model CoNi alloys from the free energy difference between fcc and hcp phases in Fig. 5. Here we further discuss and validate the temperature-dependence of SFE from the axial-next-nearest neighbor-Ising (ANNNI) model [7]. The model uses the free energy of different stacking sequences, here we use the free energy of hcp, fcc and double hcp (dhcp) structures obtained from the finite temperature free energy is the methods section. The first and second-order ANNNI estimation for

SFE [7] are

$$\begin{aligned}\gamma_1(P,T) &= \frac{2\big[F_{hcp}(V_{fcc},T) - F_{fcc}(V_{fcc},T)\big]}{A_{fcc}(T)} \\ \gamma_2(P,T) &= \frac{F_{hcp}(V_{fcc},T) - 2F_{dhcp}(V_{fcc},T) - 3F_{fcc}(V_{fcc},T)}{A_{fcc}(T)}\end{aligned} \tag{9}$$

where F_i represents the Helmholtz free energy of the structure i, $A_{fcc}(T) = \sqrt{3}/4[a(T)]^2$ is the interface area estimated from fcc phase, and $a(T)$ is the temperature-dependent lattice constant. The results for single element metal and the CoNi alloys are plotted in Supplementary Figure 4. There is no drastic difference in first and second-order SFE estimation in the current potential. For pure Ni and Co, the second-order ANNNI model SFE changes from 125.7 to 105.9 mJ/m^2, and from -20.1 to -6.1 mJ/m^2 between 78 to 1000 K, respectively. For the alloys, $Co_{70}Ni_{30}$ alloy changes from 20.4 to 16.9 mJ/m^2, $Co_{90}Ni_{10}$ alloy changes from -7.0 to -3.1 mJ/m^2, while $Co_{85}Ni_{15}$ remains nearly zero. Within the temperature range, the differences for alloys are small compared to the pure metals. Thus the approximation that SFE remains constant as a function of temperature for this model system remains valid.

SUPPLEMENTARY NOTE 3: CONVERGENCE OF DECORRELATION FORCE

Supplementary Supplementary Figure 5 presents the decorrelation force convergence vs dislocation line length. With the dimensions along the glide direction (x) and out of plane direction (y) kept as constant, we vary the dislocation line length (z) to be 69, 518, 813, and 1200 Å. The simulation details are described in the methods section. In solid solution alloys, a long dislocation will naturally adapt to a wavy configuration that lowers the total energy [8, 9]. The line tension and solute-dislocation interaction will determine the most stable configuration, called dislocation roughening [9]. As an applied stress is exerted on the dislocation, the dislocation moves through the fcc solid solution matrix and experience lattice friction plus the random configuration fluctuations. Due to the statistical nature, we expect that the dislocation line needs to exceed a certain length to show the average resistance due to line tension and solute-interaction combined. When the dislocation line length is too short, the line tension on the dislocation enforces it to move as a straight line. The total friction force to the dislocation motion will be larger. This problem can be solved if we consider a long enough dislocation line for different concentrations. The convergence results for critical dislocation line length is shown in Supplementary Figure 5.

SUPPLEMENTARY NOTE 4: SOLUTE-DISLOCATION INTERACTION ENERGY CALCULATIONS IN FCC CO

We first introduced a full dislocation in the fcc Co simulation cell as described in the methods section, where the cell was oriented along $x = [1\bar{1}0]$, $y = [111]$ and $z = [\bar{1}\bar{1}2]$ directions respectively. The simulation cell size was $2202 \times 2083 \times 87$ Å in x, y and z directions and consists of 35,904,000 atoms. The energy is partially minimized and stopped when the two partial dislocations are 653 Å apart, then we drew a $\Delta x = \pm 360$ Å squared region centered around one selected partial dislocation core that is still subject to minimization, and froze the rest of the simulation cell including the other partial dislocation. Since the solute-dislocation interaction energy changes are small but important, we used the steepest descend method for a more robust minimization. Under the new fixed boundary conditions, the free moving partial dislocation core is about 73 Å away from the fixed perfect fcc region after minimization before inserting any solutes. We then replaced one Co atom at selected position (x_i, y_i) with a Ni solute and computed the total energy. The solute-dislocation interaction energy is obtained by[10]

$$U(x_i, y_i) = E_{disl+solute}(x_i, y_i) - \bar{E}_{\text{disl+solute in bulk}}, \tag{10}$$

where $E_{\text{disl+solute}}(x_i, y_i)$ is the energy of the simulation cell with a solute at position (x_i, y_i), and the $\bar{E}_{disl+\text{solute in bulk}}$ is the energy of the dislocation in pure Co with a solute in the bulk fcc region that is far away from the core. This reference energy is averaged over 6 different solute sites in the bulk region, the reference positions are randomly selected outside of a circled region (radius ≈ 240 Å) centered on the partial dislocation. We have calculated 545 solute sites near and in the partial dislocation to construct the whole interaction energy map.

SUPPLEMENTARY REFERENCES

[1] DJ Bacon, DM Barnett, and Ronald Otto Scattergood. Anisotropic continuum theory of lattice defects. *Progress in Materials Science*, 23:51–262, 1980.

[2] G Laplanche, M Schneider, F Scholz, J Frenzel, G Eggeler, and J Schreuer. Processing of a single-crystalline CrCoNi medium-entropy alloy and evolution of its thermal expansion and elastic stiffness coefficients with temperature. *Scripta Materialia*, 177:44–48, 2020.

[3] Eddy Aerts, Pierre Delavignette, R Siems, and Severin Amelinckx. Stacking fault energy in silicon. *Journal of Applied Physics*, 33(10):3078–3080, 1962.

[4] Zhenggang Wu, Hongbin Bei, George M Pharr, and Easo P George. Temperature dependence of the mechanical properties of equiatomic solid solution alloys with face-centered cubic crystal structures. *Acta Materialia*, 81:428–441, 2014.

[5] Wataru Shinoda, Motoyuki Shiga, and Masuhiro Mikami. Rapid estimation of elastic constants by molecular dynamics simulation under constant stress. *Physical Review B*, 69(13):134103, 2004.

[6] John R Ray. Elastic constants and statistical ensembles in molecular dynamics. *Computer physics reports*, 8(3):109–151, 1988.

[7] Xi Zhang, Blazej Grabowski, Fritz Körmann, Andrei V Ruban, Yilun Gong, Roger C Reed, Tilmann Hickel, and Jörg Neugebauer. Temperature dependence of the stacking-fault gibbs energy for al, cu, and ni. *Physical Review B*, 98(22):224106, 2018.

[8] Céline Varvenne, Aitor Luque, and William A Curtin. Theory of strengthening in fcc high entropy alloys. *Acta Materialia*, 118:164–176, 2016.

[9] Gerard Paul M Leyson, William A Curtin, Louis G Hector Jr, and Christopher F Woodward. Quantitative prediction of solute strengthening in aluminium alloys. *Nature materials*, 9(9):750, 2010.

[10] Gerard Paul M Leyson, LG Hector Jr, and William A Curtin. Solute strengthening from first principles and application to aluminum alloys. *Acta Materialia*, 60(9):3873–3884, 2012.
